# Plain radiographic indices are reliable indicators for quantitative bone mineral density in male and female patients before total hip arthroplasty

**DOI:** 10.1038/s41598-023-47247-w

**Published:** 2023-11-14

**Authors:** Sebastian Rohe, Sabrina Böhle, Georg Matziolis, Benjamin Jacob, Steffen Brodt

**Affiliations:** Orthopaedic Department of the Waldkliniken Eisenberg, Professorship of the University Hospital Jena, Campus Waldkliniken Eisenberg, Klosterlausnitzer Straße 81, 07607 Eisenberg, Germany

**Keywords:** Risk factors, Medical research, Musculoskeletal system

## Abstract

Osteoporosis is underdiagnosed in patients undergoing total hip arthroplasty (THA). Bone mineral density measurement by dual-energy X-ray absorptiometry (DXA) is the gold standard, but indices on plain hip radiographs also seemed to be reliable screening tools in female or Asian ethnicities in previous studies. Given the lack of knowledge about male patients and Caucasian ethnicities, this study was conducted to evaluate plane hip radiographic indices as a screening tool for osteopenia and osteoporosis in Caucasian female and also male patients before undergoing THA. A retrospective analysis of 216 elderly patients with pre-existing DXA before hip arthroplasty was performed and four indices were calculated on plain hip radiographs: Canal-Flare-Index (CFI), Canal-Calcar-Ratio (CCR), Canal-Bone-Ratio (CBR) 7 and 10 cm below the lesser trochanter. They were correlated with femoral neck DXA T-scores by Pearson’s correlation and intraclass correlation coefficient, and a ROC analysis was performed. A total of 216 patients (49.5% male) were included. CBR-7 and -10 were highly correlated (p < 0.001) with femoral neck T-score in males (Pearson’s correlation CBR-7 r = − 0.60, CBR-10 r = − 0.55) and females (r = − 0.74, r = − 0.77). CBR-7 and -10 also showed good diagnostic accuracy for osteoporosis in the ROC analysis in males (CBR-7: AUC = 0.75, threshold = 0.51; CBR-10: 0.63; 0.50) and females (CBR-7: AUC = 0.87, threshold = 0.55; CBR-10: 0.90; 0.54). Indices such as the Canal Bone Ratio (CBR) 7 or 10 cm below the lesser trochanter on plain hip radiographs are a good screening tool for osteopenia and osteoporosis on plain hip radiographs and can be used to initiate further diagnostics like the gold standard DXA. They differ between male and female patients.

## Introduction

Osteoporosis has become a growing problem in recent years as the number of elderly patients has increased. Furthermore, reflecting the demand for an active lifestyle even in older patients, the number of total hip arthroplasties (THA) implanted is increasing. In patients with end-stage osteoarthritis, osteoporosis was found in 18%^1^. In addition to an increased risk of fracture, osteoporosis impairs stable fixation of implants, resulting in a high risk of failure despite correct reconstruction and implant positioning^2,3^. Decreased bone mineral density (BMD) due to low bone mineralization is one aspect of impaired bone quality in osteoporosis. BMD can be measured by dual-energy X-ray absorptiometry (DXA), which remains the gold standard for diagnosing and monitoring osteoporosis^4–7^. Another diagnostic tool to classify bone quality was quantitative computed tomography (QCT), which is based on the principle of volumetric analysis of bone structure. By estimating cortical and cancellous bone in a three-dimensional model, the amount of bone could be more accurately estimated. Disadvantages of this technique include high cost, limited availability, and much higher radiation exposure. QCT may be beneficial only in individual situations and for investigational purposes, but is not recommended for screening^8,9^. Radiographs of the hip are readily and inexpensively available in patients being prepared for hip arthroplasty due to osteoarthritis and could be used for osteoporosis screening, e.g. in preoperative planning of THA by surgeons or by artificial intelligence and machine deep learning^10^.

In 1960, Barnett and Nordin investigated the relationship between cortical thickness and BMD and introduced the metacarpal index^11^. In 1970, Singh showed that a thinning of the trabecular pattern in the proximal femur on plain radiographs may indicate abnormal bone loss and thus osteoporosis^12–15^. Shankar et al. investigated the correlation between femoral geometry on plain radiographs and CT-Scans with DXA and showed a correlation of bone morphologic parameters and the DXA value^16,17^. Another method to classify femoral morphology and secondary bone quality based on plain radiographs of the hip was presented by Dorr et al. They identified radiographic parameters and indices that correlated with morphology and bone quality and validated them by histologic examination^18^. The cortical thickness index (CTI) as radiographic parameter showed in further studies a significant correlation with BMD measured by DXA in female patients with coxarthrosis who were scheduled for THA^19^. Similarly, Yeo et al. demonstrated a correlation between CTI and canal-calcar ratio (CCR) on plain radiographs of the hip with BMD and the presence of osteoporosis in 112 elderly patients after proximal femoral fractures^20^. However, a limitation of this study is the small number of male patients (n = 21) and the inclusion of patients with femoral neck fractures only. Similarly, Liu et al. demonstrated the reliability of the canal bone ratio (CBR) as an indicator of femoral osteopenia on plain radiographs of the hip in 81 patients, with the limitations of not differentiating between gender and including only Asian patients^21^. Ethnic diversity of osteoporosis was already shown with different prevalences of osteoporosis in Caucasian, Asian or African ethnicities^22,23^. Furthermore, different thresholds for diagnosing a osteoporosis especially in Asian population are discussed^24^.

Artificial intelligence (AI) is also an emerging field in medicine, particularly in the interpretation of plain radiographs. Recent studies have applied machine deep learning to hip plain radiographs to measure implant position^25^ and indices for hip dysplasia^26^. Deep learning algorithms are also being developed for digital preoperative planning of THA^10^. Thresholds for indices are necessary to train the AI to identify patients at risk for osteoporosis, so that it could recommend further diagnostics and e.g. cemented procedures for THA.

This study therefore aimed to establish thresholds based on preoperative plain radiographs of the hip, also in Caucasian patients and with a larger number of male patients, to differentiate between patients who have clinically relevant osteoporosis and should be referred for further diagnostics such as DXA and recommended for cemented THA.

## Materials and methods

This is a retrospective study of male and female Caucasian patients treated with unilateral THA for primary osteoarthritis who underwent preoperative dual-energy X-ray absorptiometry and standardized plain radiographs of the hip within one year. The study was approved by the local ethics committee (Reg. No. 2022-2814-Daten). All procedures were performed between 2013 and 2022 in an orthopedic maximum care hospital in accordance with relevant guidelines/regulations, especially the Declaration of Helsinki. Informed consent was obtained from all patients. Inclusion criteria were age over 18 years, Caucasian ethnicity, DXA and plain radiograph within one year, no previous osteoporotic fracture, no osteo-metabolic medication. Exclusion criteria were previous femoral trauma, femoral head collapse, and radiographic tumors, rheumatoid arthritis, corticosteroid therapy, severe kidney diseases, severe chronic liver diseases, hypo- and hyperthyroidism, bad quality of plain radiograph, shown femoral length less than 12 cm, missing reference sphere, time discrepancy of DXA and plain radiograph over 1 year. DXA measurement of the proximal femoral neck was performed using the Hologic Horizon DXA system (Hologic GmbH, 65205 Wiesbaden-Nordenstadt, Germany). Osteoporosis was considered if the T-score was − 2.5 or less, and osteopenia was considered if the T-score was between − 1.0 and − 2.5^4,27^. Standardized plain radiographs of the hips were obtained with the patients in the supine position and the lower extremities internally rotated 15°, centered on the line connecting the midpoint of the anterior superior iliac spine and the pubic symphysis using a Carestream DRX-Compass-System (Carestream Health Inc, Rochester, NY 14608, USA).

### Measurement

**Figure 1:**
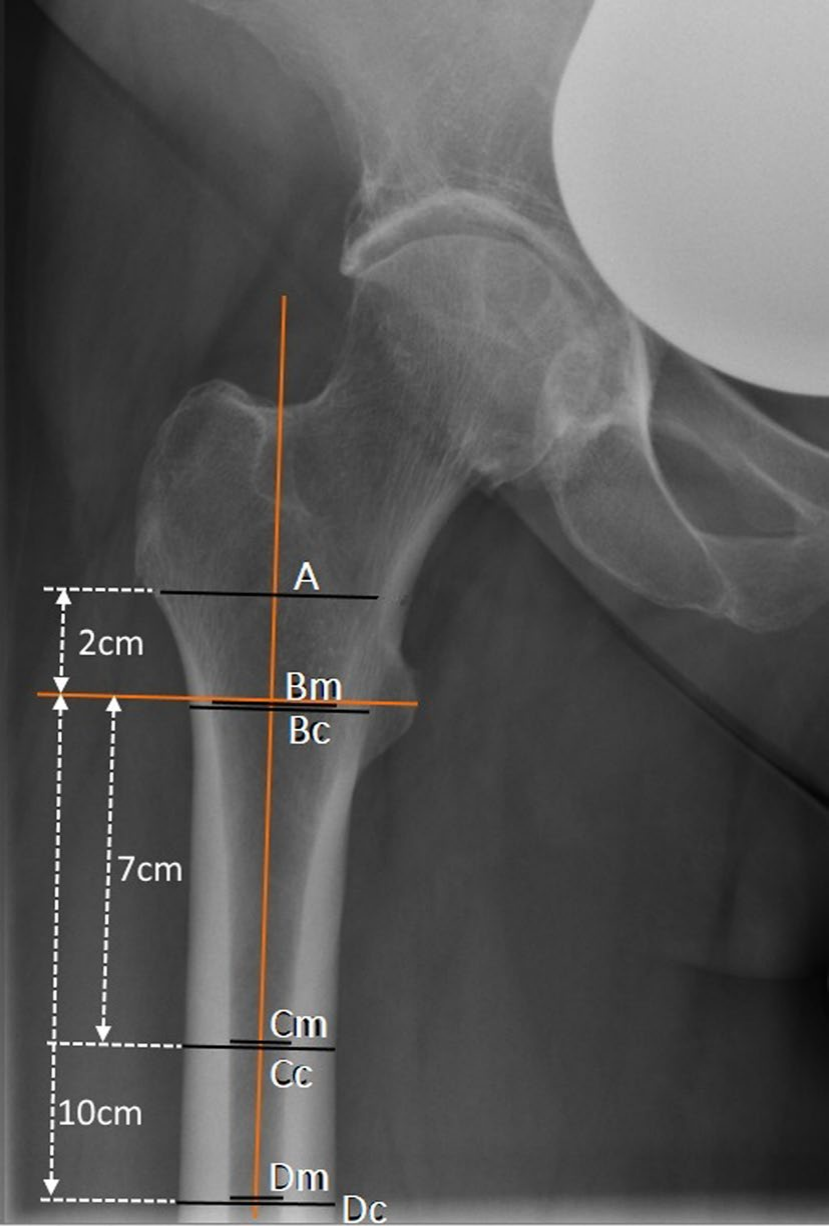
Measurement of the indices on plain ap radiographs: canal-bone-ratio CBR-7 cm (= Cm/Cc) and CBR-10 cm (= Dm/Dc), canal-calcar ratio CCR (= Dm/Bm); canal flare index CFI (= A/Dm).

Digitally stored plain hip radiographs were normalized using a calibrated 25 mm sphere in the PACS system IMPAX EE R20 XVII (Agfa HealthCare Europe, 2640 Mortsel, Belgium). The radiographic indices shown in Fig. 1 were then determined by an orthopedic surgeon (Fig. 1). Yeung and Liu et al. already showed a good intra- and interobserver reliability of these indices^21,28^. First, the femoral shaft axis was determined and a reference line perpendicular to the femoral shaft was drawn through the center of the lesser trochanter. Lines were then measured 2 cm above and 7 and 10 cm below this reference^28^. At this height, the medulla-cortical ratio was measured as the canal-bone ratio CBR-7cm (= Cm/Cc) and CBR-10 cm (= Dm/Dc)^28^. The canal-calcar ratio CCR (= Dm/Bm) according to Dorr^18^ and the canal flare index CFI (= A/Dm)^21,28^ were also determined (Fig. 1). Measurements were performed using the PACS system IMPAX EE R20 XVII (Agfa HealthCare Europe, 2640 Mortsel, Belgium).

### Statistical analysis

Data management was performed using Microsoft Excel 365 (Microsoft, Redmond, USA) and statistical analysis was performed using SPSS 28 (IBM, Armonk, NY, USA). All quantitative data are presented as mean ± standard deviation (SD). Indices were correlated using Pearson's correlation coefficients (± 1.0 to 0.9 very high correlation, ± 0.9 to 0.7 high correlation; ± 0.7 to 0.5 moderate correlation; ± 0.5 to 0.3 low correlation; ± 0.3 to 0.0 negligible correlation) and a two-sided mixed intraclass correlation coefficient (values < 0.5 indicate poor, 0.5 to 0.75 moderate, 0.75 to 0.9 good, > 0.90 excellent correlation)^29–32^. Normal distribution of the data was tested with Shapiro–Wilk’s test. Student’s t-test was used for normal distributed data and Mann–Whitney-*U*-test for non-normal distributed data to compare the indices of the non-osteopenia and osteopenia group. Subsequently, receiver operating characteristic (ROC) curve analysis was used for testing the diagnostic value of the indices for osteopenia (T-score ≤ -1.0) and osteoporosis (T-score ≤ -2.5) and the area under the curve (AUC) was calculated to assess the reliability^21^. Test accuracy was considered as follows: AUC between 0.90 and 1.00 = excellent discrimination ability, AUC between 0.80 and 0.90, 0.70 and 0.80, 0.60 and 0.70 and 0.50 to 0.60 = good, fair, poor and fail discrimination ability, respectively^33^. The cut-off values were determined when the Youden index was at its maximum^34^. Differences were considered to be statistically significant at p < 0.05.

### Ethical approval

Ethical approval was done by the local Ethics Committee of University Jena in view of the retrospective nature of the study. All procedures being performed were part of the routine care (RNr. 2022-2814-Daten) and all research was performed in accordance with relevant guidelines/regulations especially the Declaration of Helsinki.

### Informed consent

Informed consent was obtained from all individual participants included in the study.

## Results

**Figure 2:**
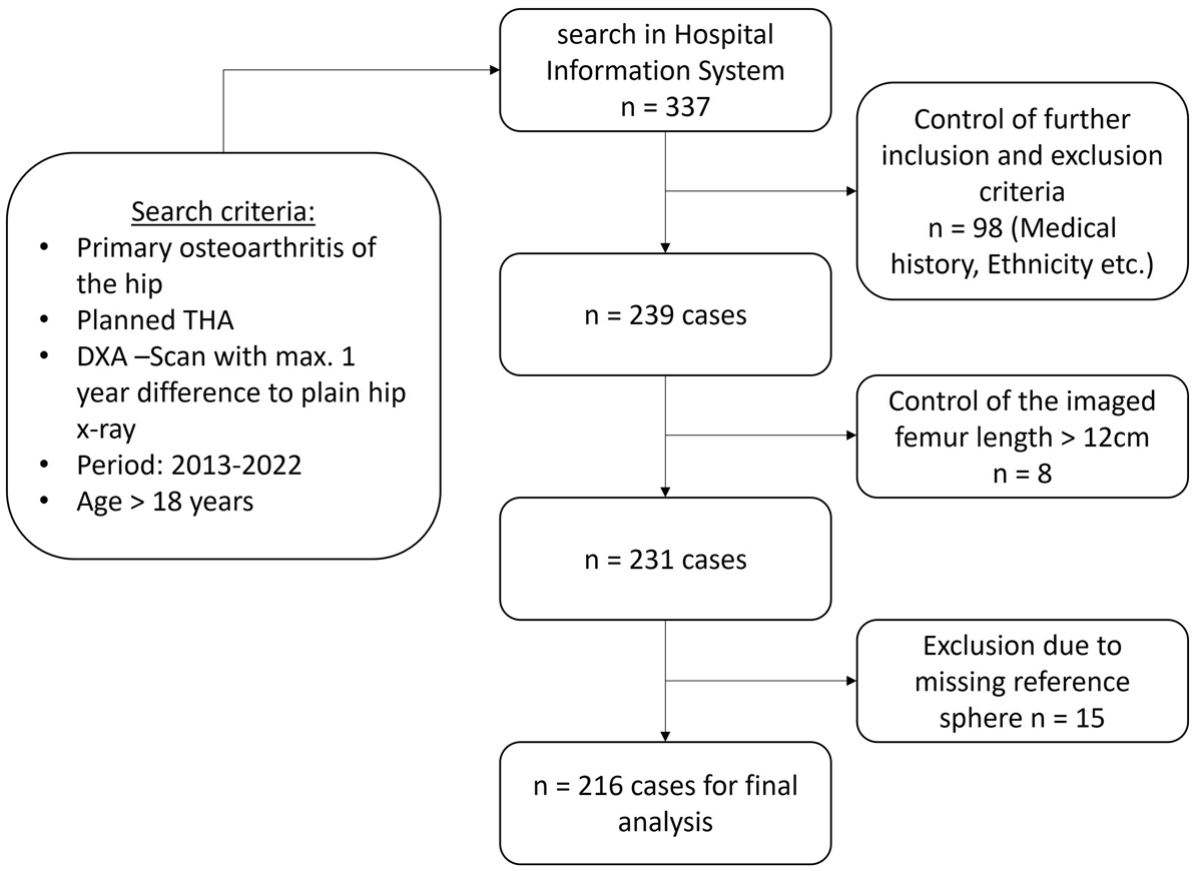
Flow chart of case selection, *DXA* dual-energy X-ray absorptiometry, *THA* Total hip arthroplasty.

**Figure 3:**
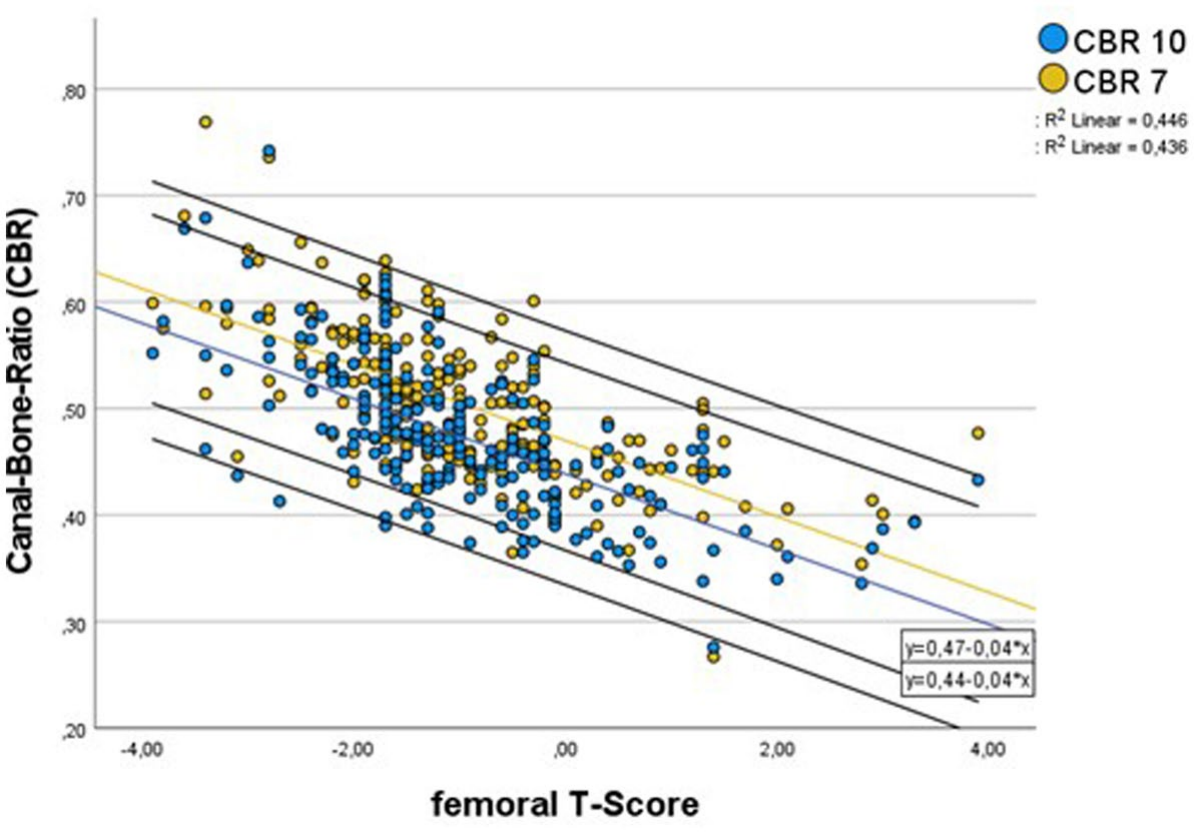
Scatter Plot of the correlation of CBR-7 and CBR-10 to the proximal femoral neck T-score, linear trend-line with 95%-confidence interval.

After evaluation of the inclusion and exclusion criteria, 216 patients with 216 plain radiographs were finally analyzed (Fig. 2) and the indices were measured (Fig. 1). The demographic and anthropometric data of the patients were shown in Table 1. The male collective was significantly younger, taller and had a higher T-score. Body mass index (BMI) did not show significant differences between male and female patients. Pearson’s correlations and interclass correlation coefficients of the four measured indices were shown in Table 2. For Pearson’s correlation to the proximal femoral neck T-Score, CBR-7 and CBR-10 showed the best correlation in male and female patients (Fig. 3), while CCR showed only a poor correlation and CFI showed a good correlation in female but only a poor correlation in male. Except for CCR in males, all indices showed a significant correlation to the proximal femoral neck T-Score (Table 2).

**Table 1:** Patients characteristics.

	Total	Male	Female	p-Value
Number (%)	216	107 (49,5)	109 (50,5)	
Age (years); (min–max)	69.77 ± 10.13 [32–96]	67.64 ± 10.43 [32–86]	71.86 ± 9.41 [46–92]	0.002
Age > 60 years	180	84	96	
Height (cm)	169.87 ± 9–67	176.33 ± − 8.33	163.52 ± 5.99	< 0.001
BMI (kg/m^2^)	27.58 ± 4.13	27.83 ± 4.10	27.33 ± 4.17	0.372
Proximal femoral neck T-score	− 0.97 ± 1.31	− 0.53 ± 1.39	− 1.39 ± 1.02	< 0.001
Right femora (%)	49.1	49.5	48.6	0.894

*BMI* Body-Mass-Index.

**Table 2:** Correlation of the indices to the T-score.

Indices	Pearson’s correlation [rho r] (p-value)			Intraclass-correlation-coefficient (95%-CI)		
	Total	Male	Female	Total	Male	Female
Calcar-canal ration (CCR)	− 0.232 (< 0.001)	− 0.156 (0.108)	− 0.386 (< 0.001)	0.145 (− 0.118–0.346)	0.100 (− 0.320–0.386)	0.205 (− 0.162–0.456)
Calcar-flare-Index (CFI)	0.442 (< 0.001)	0.332 (< 0.001)	0.625 (< 0.001)	0.492 (0.335–0.611)	0.405 (0.127–0.594)	0.567 (0.368–0.704)
Canal-bone-ratio at 7 cm (CBR-7)	− 0.668 (< 0.001)	− 0.602 (< 0.001)	− 0.739 (< 0.001)	0.781 (0.714–0.833)	0.752 (0.636–0.831)	0.767 (0.660–0.841)
Canal-bone-ratio at 10 cm (CBR-10)	− 0.660 (< 0.001)	− 0.553 (< 0.001)	− 0.767 (< 0.001)	0.776 (0.707–0.828)	0.711 (0.576–0.803)	0.792 (0.696–0.857)

*95% CI* 95% confidence interval.

**Figure 4:**
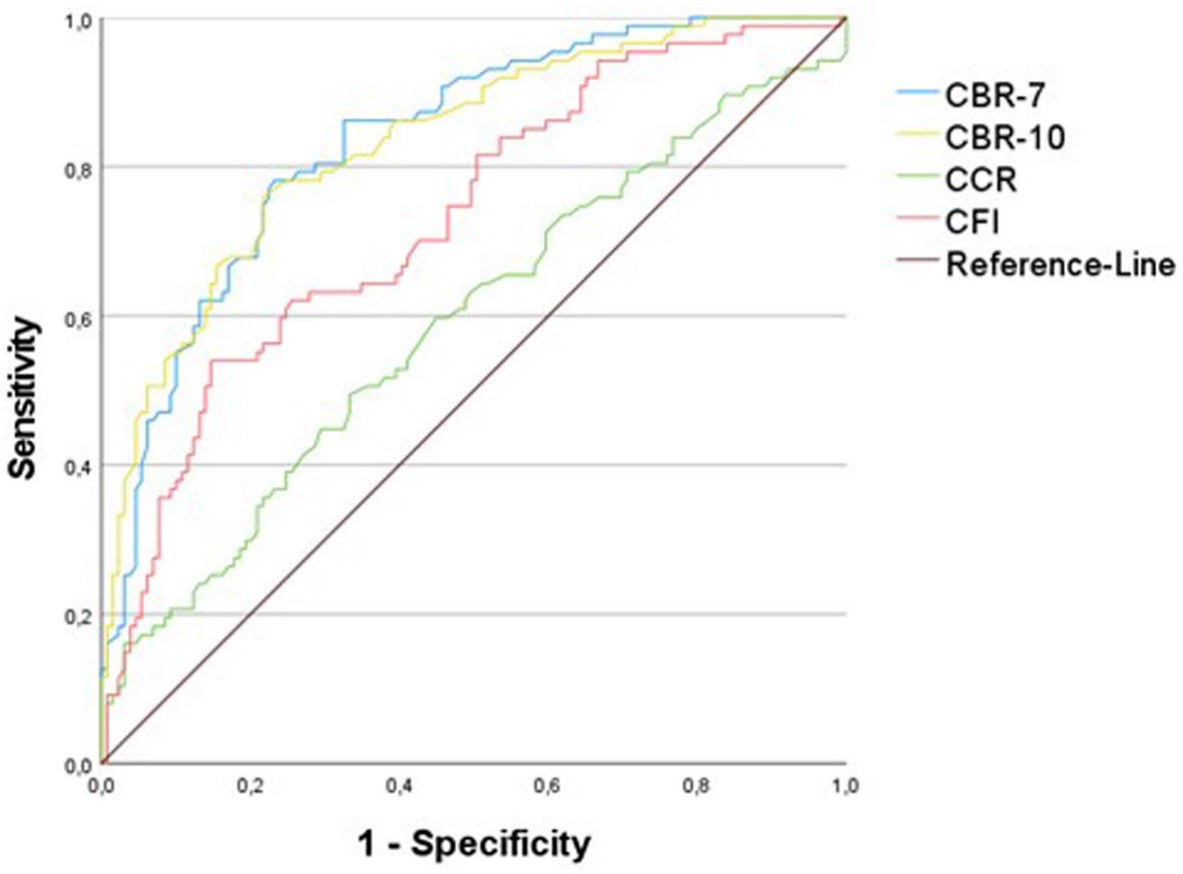
ROC of the Canal-Bone-Ratio-7 (CBR-7), Canal-Bone-Ratio-10 (CBR-10), Canal-Calcar-Ratio (CCR), Canal-Flare-Index (CFI) for diagnosing osteopenia (T-score ≤ − 1.0).

**Figure 5:**
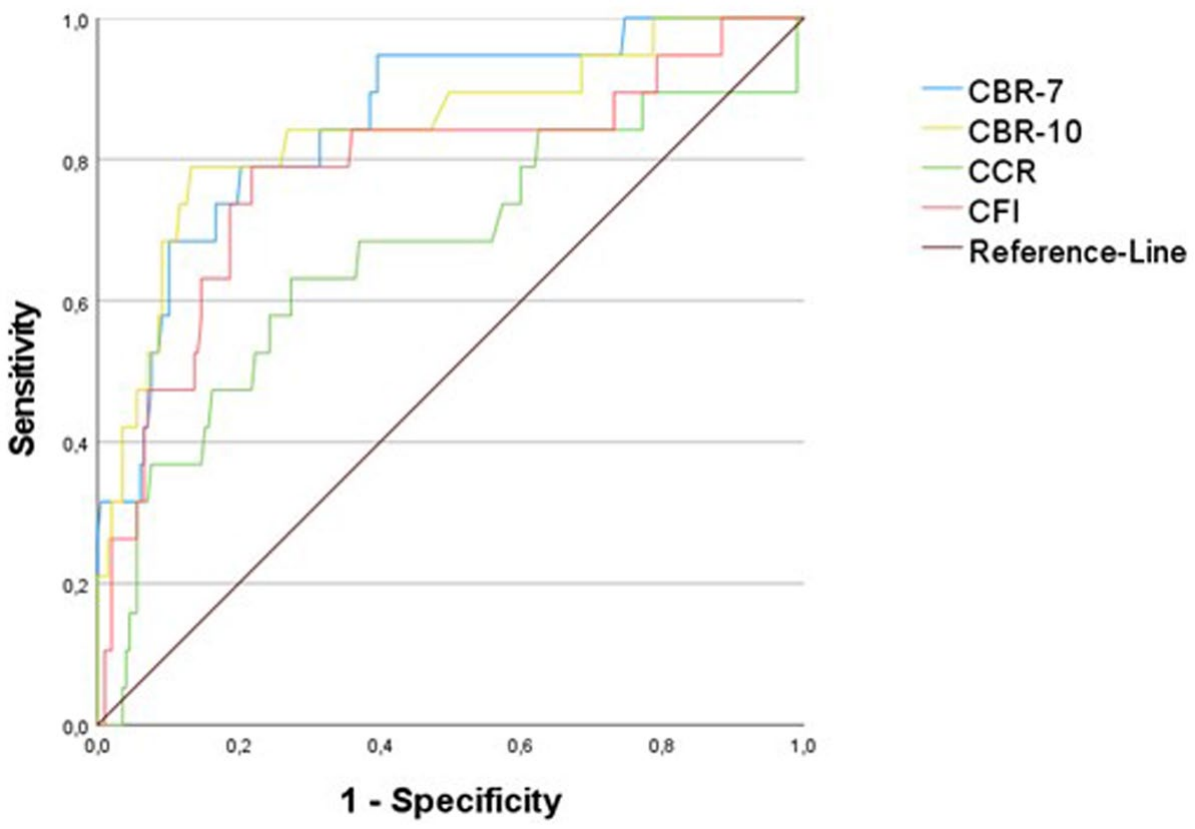
ROC of the Canal-Bone-Ratio-7 (CBR-7), Canal-Bone-Ratio-10 (CBR-10), Canal-Calcar-Ratio (CCR), Canal-Flare-Index (CFI) for diagnosing osteoporosis (T-score ≤ − 2.5).

The Receiver-Operation-Curve (ROC) analysis for the determination of osteopenia showed good discrimination for the CBR-7 and CBR-10 in all and female patients, as well as a fair correlation in males. The CFI also showed good discrimination for osteopenia in all and female patients. The CCR showed poor to no discrimination in the groups. Regarding osteoporosis, the CBR-7 and CBR-10 again showed a good ability for discrimination in male and female patients. Male patients showed only fair discrimination for the CBRs. The CFI showed a good discriminatory ability in females, a fair discriminatory ability in all patients, and a poor discriminatory ability in males, while the CCR showed a fair discriminatory ability, a poor discriminatory ability, and no discriminatory ability (Table 3). The ROC and area under the curve analysis were shown for osteopenia (Fig. 4) and osteoporosis (Fig. 5). After determining the most accurate threshold in each case using the Youden index, the sensitivity and specificity for the indices were obtained (Table 4). The threshold ratios in male, female and all patients were significantly different in CBR-7 and CBR-10 (p < 0.001).

**Table 3:** ROC-Analysis of the indices for osteopenia and osteoporosis.

	Osteopenia—AUC (95%-CI)			Osteoporosis—AUC (95%-CI)		
	Total	Male	Female	Total	Male	Female
CCR	0.589 (0.510–0.668)	0.496 (0.365–0.626)	0.665 (0.564–0.766)	0.668 (0.521–0.814)	0.385 (0.014–0.756)	0.752 (0.608–0.896)
CFI	0.730 (0.662–0.798)	0.655 (0.544–0.766)	0.798 (0.715–0.880)	0.783 (0.658–0.907)	0.544 (0.259–0.828)	0.861 (0.742–0.980)
CBR-7	0.835 (0.781–0.888)	0.771 (0.679–0.863)	0.875 (0.807–0.942)	0.854 (0.765–0942)	0.748 (0.601–0.894)	0.865 (0.758–0.971)
CBR-10	0.832 (0.777–0.887)	0.734 (0.631–0.837)	0.885 (0.823–0.948)	0.844 (0.738–0.950)	0.631 (0.373–0.889)	0.901 (0.809–0.994)

*AUC* Area-Under-the-Curve, *CCR* Canal-Calcar-Ratio, *CFI* Canal-Flare-Index, *CBR-7* Canal-Bone-Ratio at 7 cm, *CBR-10* Canal-Bone-Ratio at 10 cm, *CI* Confidence-Interval.

**Table 4:** Calculated most accurate threshold ratio via Youden-Index.

Index		Osteopenia				Osteoporosis			
		Ratio	Sens	Spec	YI	Ratio	Sens	Spec	YI
CBR-7	Total	0.5105	0.782	0.767	0.549	0.5465	0.789	0.797	0.586
CBR-10		0.4755	0.759	0.783	0.542	0.5350	0.789	0.868	0.657
CCR		0.5320	0.494	0.667	0.161	0.5545	0.632	0.726	0.357
CFI		3.1710	0.540	0.853	0.393	3.1075	0.789	0.782	0.571
CBR-7	Male	0.4885	0.774	0.697	0.472	0.5115	1.000	0.650	0.650
CBR-10		0.4725	0.645	0.724	0.369	0.5020	0.500	0.786	0.286
CCR		0.7015	0.129	0.987	0.116	0.5655	0.500	0.728	0.228
CFI		3.1710	0.484	0.816	0.300	2.9725	0.500	0.825	0.325
CBR-7	Female	0.5120	0.839	0.830	0.669	0.5465	0.933	0.766	0.699
CBR-10		0.4755	0.857	0.811	0.668	0.5350	0.933	0.830	0.763
CCR		0.5480	0.464	0.811	0.276	0.5875	0.533	0.904	0.438
CFI		3.3055	0.696	0.811	0.508	3.1075	0.867	0.798	0.665

*Sens.* Sensitivity, *Spec.* Specificity, *YI* Youden-Index, *CCR* Canal-Calcar-Ratio, *CFI* Canal-Flare-Index, *CBR-7* Canal-Bone-Ratio at 7 cm, *CBR-10* Canal-Bone-Ratio at 10 cm.

## Discussion

This study investigates the correlation between indices on standardized preoperative plain radiographs of the hip and the proximal femoral neck T-score, measured by DXA, in a Caucasian population with a high number of male patients depending on sex. Previous studies often had a low number or lack of discrimination of male patients^19,20^, looked at Asian collectives^19^, or fractured femora with a questionable lack of standardized radiographs^20^.

An easily accessible screening tool for osteopenia or osteoporosis using plain hip radiographs is of great importance for the orthopedic hip surgeons to avoid potential surgical risks while performing total hip arthroplasty due to osteopenia and osteoporosis^1–3^. This study was able to show a good correlation of the CBR-7 and CBR-10 to the proximal femoral neck T-score (Table 2), generally confirming the results of Young et al. and Liu et al.^21,28^, while CCR and CFI had only a poor to fair correlation^21^. The sub-analysis of CBR-7 and CBR-10 concerning sex showed a good correlation in female patients and a slightly worse correlation in male patients in this collective. Yeo et al. also described a good correlation in 91 female patients, but a lack of correlation between the cortical thickness index and the T-score in their 21 male patients^20^. This could be explainable by the low number of male patients in their study or by the different pathways of bone mineral density loss in postmenopausal and senile women and senile men^35–38^. Women are more likely to initially suffer from type I primary osteoporosis (postmenopausal) with initial cancellous accentuated high turnover bone loss. In contrast, men suffer from senile primary osteoporosis type II with cortical accentuated low turnover bone loss^38^, which may explain the discrepancies between male and female CBR to BMD correlations.

Contrary to Yeo et al. and Liu et al. this study could report a linear correlation of the CCR (r = − 0.232), which confirmed the results of Yeung at el. (r = − 0.34)^20,21,28^. But this correlation was not as significant as for the CBR (Table 2) and only with a negligible to low correlation^32^. For the CFI Liu et al. (r = 0.36) and Yeung et al. (r = 0.46) confirmed the linear correlation to the T-Score reported in this study (r = 0.442) (Table 2). But again with a weak correlation^32^. Therefore, these indices could not be recommended for screening for osteopenia or osteoporosis.

In terms of diagnostic accuracy CBR-7 (AUC: 0.835, threshold ratio: 0.5105) and CBR-10 (AUC: 0.832, threshold ratio: 0.4725) had the highest AUC in both female and male patients (Table 3). The most recent study by Liu et al. analyzed 81 patients (49 non-osteopenia) and showed for the CBR-7 an AUC of 0.7688 (95%-CI: 0.6670 to 0.8706) and a threshold ratio for osteopenia of 0.4165 and for the CBR-10 an AUC of 0.8207 (95%-CI: 0.7299 to 0.9115) and a threshold ratio of 0.3805 calculated by Youden-index without sex discrimination^21,34^. Yeung et al. reported an AUC for detecting osteoporosis with CBR at 10 cm distal to the lesser trochanter of 0.84 (95%-CI: 0.72 to 0.95) and a threshold ratio of 0.49^28^. These values were close to the thresholds reported in this study. In male patients with the most accurate indices CBR-7 and CBR-10, this study reported a threshold ratio of 0.49 and 0.47 for osteopenia and 0.51 and 0.50 for osteoporosis, respectively. These thresholds are slightly lower than in female patients with threshold ratios of 0.51 and 0.48 for osteopenia and 0.55 and 0.54 for osteoporosis, respectively. This implies a wider medullary space or a thinner cortex in osteopenia and osteoporotic women than in men. Regarding the cortical thickness index (CTI), Nguyen et al. previously described a difference between male and female patients of Asian ethnicity and confirmed a sex difference in cortical indices, but did not investigate CBR^39^. This difference could also be explained by the different type of bone loss in male and female patients^35–38^.

In Summary, this study showed that cortical indices such as CBR-7 and CBR-10 had a moderate to high linear correlation with proximal femoral neck T-score and were a good tool to screen for osteopenia (sens.: 78%, 76%; spec.: 77%;78%) or osteoporosis (sens.: 79%, 79%; spec.: 80%;87%) with a significant but marginal difference in male to female patients. According to an easy-to-use parameter, we recommend a gender-independent threshold ratio of CBR-7 or CBR-10 of 0.49 or 0.47 for osteopenia and 0.51 or 0.50 for osteoporosis to consider further diagnostics such as DXA.

Looking ahead to the increasing integration of artificial intelligence in the interpretation of plain radiographs, including hip radiographs especially for planning THA, these indices could be used to assess osteopenia or osteoporosis preoperatively and optimize surgical preparation prior to THA^10^.

There were several limitations to this study. Despite standard positioning during radiography, femoral rotation was sometimes limited due to massive osteoarthritis, resulting in potential rotational failure of the measurement and also the long study period could be leaded to diversity in X-ray images. Second, we did not perform an intra- or interobserver correlation test because this had been well studied in previous studies and has shown good reliability^21,28,39^. Third, we studied only Caucasian patients, so these data may not be representative of other ethnicities.

Despite a good correlation between CBR and T-score, bone densitometry by DXA is still the gold standard and cannot be replaced by plain radiographic indices. Nevertheless, the easy to perform measurement can serve as a quick criterion and screening tool especially in the context of artificial intelligence and machine learning to initiate further diagnostic procedures such as DXA and thus protect patients from osteoporosis-related risks, e.g. intraoperative fractures^2,3^. Its use in THA planning is also becoming more interesting with the use of deep learning algorithms to identify patients at risk^10^, so that they can be referred for further diagnostic and surgical procedures can be customized e.g. cemented THA.

## Conclusion

Indices such as the Canal Bone Ratio (CBR = medullary diameter/cortical diameter) 7 or 10 cm below the lesser trochanter on plain radiographs of the hip are a good screening tool for osteopenia and osteoporosis and for deciding whether further diagnostic workup is needed. They differ slightly between male and female patients. In the future, the calculated values could be used by AI planning algorithms to recommend additional preoperative DXA to individualize the surgical procedure.

## Data Availability

The datasets used and/or analysed during the current study are available from the corresponding author on reasonable request.
